# The Epidemiology of Hip and Major Osteoporotic Fractures in a Dutch Population of Community-Dwelling Elderly: Implications for the Dutch FRAX® Algorithm

**DOI:** 10.1371/journal.pone.0143800

**Published:** 2015-12-03

**Authors:** Corinne Klop, Paco M. J. Welsing, Hubert G. M. Leufkens, Petra J. M. Elders, Jetty A. Overbeek, Joop P. van den Bergh, Johannes W. J. Bijlsma, Frank de Vries

**Affiliations:** 1 Utrecht Institute for Pharmaceutical Sciences, Division of Pharmacoepidemiology and Clinical Pharmacology, Utrecht University, Utrecht, Netherlands; 2 Department of Rheumatology and Clinical Immunology, University Medical Center, Utrecht, Netherlands; 3 Julius Center for Health Sciences and Primary Care, University Medical Center, Utrecht, Netherlands; 4 Department of General Practice and Elderly Care, VU University Medical Centre, Amsterdam, Netherlands; 5 PHARMO Institute for Drug Outcomes Research, Utrecht, Netherlands; 6 Department of Internal Medicine, Viecuri Medical Centre, Venlo, Netherlands; 7 Department of Internal Medicine, Maastricht University Medical Centre+, Maastricht, Netherlands; 8 Biomedical Research Institute, University Hasselt, Hasselt, Belgium; 9 Department of Clinical Pharmacy and Toxicology, Maastricht University Medical Centre+, Maastricht, Netherlands; 10 MRC Lifecourse Epidemiology Unit, Southampton General Hospital, Southampton, United Kingdom; Garvan Institute of Medical Research, AUSTRALIA

## Abstract

**Background:**

Incidence rates of non-hip major osteoporotic fractures (MOF) remain poorly characterized in the Netherlands. The Dutch FRAX® algorithm, which predicts 10-year probabilities of hip fracture and MOF (first of hip, humerus, forearm, clinical vertebral), therefore incorporates imputed MOF rates. Swedish incidence rate ratios for hip fracture to MOF (Malmo 1987–1996) were used to perform this imputation. However, equality of these ratios between countries is uncertain and recent evidence is scarce. Aims were to estimate incidence rates of hip fracture and MOF and to compare observed MOF rates to those predicted by the imputation method for the Netherlands.

**Methods:**

Using hospitalisation and general practitioner records from the Dutch PHARMO Database Network (2002–2011) we calculated age-and-sex-specific and age-standardized incidence rates (IRs) of hip and other MOFs (humerus, forearm, clinical vertebral) and as used in FRAX®. Observed MOF rates were compared to those predicted among community-dwelling individuals ≥50 years by the standardized incidence ratio (SIR; 95% CI).

**Results:**

Age-standardized IRs (per 10,000 person-years) of MOF among men and women ≥50 years were 25.9 and 77.0, respectively. These numbers were 9.3 and 24.0 for hip fracture. Among women 55–84 years, observed MOF rates were significantly higher than predicted (SIR ranged between 1.12–1.50, depending on age). In men, the imputation method performed reasonable.

**Conclusion:**

Observed MOF incidence was higher than predicted for community-dwelling women over a wide age-range, while it agreed reasonable for men. As miscalibration may influence treatment decisions, there is a need for confirmation of results in another data source. Until then, the Dutch FRAX® output should be interpreted with caution.

## Introduction

Osteoporotic fractures are a worldwide epidemic resulting in significant morbidity, mortality, and high health care costs [1–3]. Due to the ageing population this burden has been projected to increase greatly with an estimated number of 4.5 million fractures in Europe in 2025 [4]. It is therefore important to identify those with an increased risk of fracture to direct effective interventions.

The development of the FRAX® algorithm by the World Health Organization has led to a shift in identifying fracture risk from bone mineral density measurement towards absolute risk assessment. This algorithm is intended for primary care and incorporates clinical risk factors with or without bone mineral density (BMD) to compute the 10-year probability of hip or a major osteoporotic fracture ([MOF] first of hip, clinical spine, humerus, or forearm). It has been incorporated internationally in clinical guidelines and is frequently used with over 13 million assessments by the FRAX® webpage between 2011 and 2015 [5–9]. Since hip fracture rates do not only vary widely by age and sex but also by geographic region [2], FRAX® algorithms require country-specific fracture rates and rates for mortality. There are now 62 FRAX® algorithms available for specific countries and ethnicities. The Dutch model has become available in the year 2010 [10].

In contrast to hip fracture, country-specific data for the incidence of MOF are scarce. This is because most fractures at other sites than the hip do not require hospitalization. In the absence of such data, FRAX® algorithms incorporate imputed rates of MOF. This is performed by adopting them from a neighboring country or by assuming equal age-and-sex-specific incidence rate ratios of hip fracture to other MOFs as were observed in Malmö, Sweden [11]. There is, however, only limited evidence that supports the assumption of equal ratios between countries. And importantly, secular changes in incidence of hip and non-hip fractures over the past decade(s) may have violated this imputation method. The Dutch FRAX® algorithm has incorporated hip fracture rates from 2004/2005, and the historical Swedish data (1987–1996) was used to impute MOF incidence. Indeed, a decline in hip fracture incidence was observed in several countries [2], including Sweden [12] and the Netherlands [13], but far less is known about fractures at other sites.

We therefore aimed to estimate age-and-sex-specific incidence rates of hip and other MOFs separately (humerus, forearm, clinical spine) and as used in FRAX® (first of hip, humerus, forearm, or clinical spine) in a Dutch community-dwelling population. A second aim was to compare observed MOF rates to those predicted by the imputation method.

## Methods

### Data source

A cohort study was performed within the Dutch PHARMO Database Network [PHARMO Institute for Drug Outcome Research, www.pharmo.nl]. This network links drug dispensing records to hospital discharge records (www.dutchhospitaldata.nl), general practitioner (GP) and death registration data using probabilistic linkage [14, 15]. For the current study these data was available for approximately 660,000 community-dwelling individuals (comprising more than 4.9 million person-years of follow-up) from the Netherlands between 1 January 2002 and 31 December 2011. Primary care diagnoses are coded according to International Classification of Primary Care (ICPC) codes. Hospital records include dates of hospital admission and discharge, diagnoses, procedures and are recorded according to the International Classification of Disease, 9^th^ or 10^th^ revision codes (ICD-9 or ICD-10) [16]. High validity of hip fracture coding has been shown previously in the PHARMO record linkage system where >90% of recorded hip fractures represented true hip fractures [17]. The study was approved by the Compliance Committee of the PHARMO Institute. Patient records were anonymized and de-identified by the PHARMO Institute before providing the data to the authors for analysis.

### Study outcomes

Fractures were classified into the following categories using ICPC, ICD-9 and ICD-10 codes: hip (ICPC: L75.01, ICD-9: 820, ICD-10: S72.0, S72.1, S72.2), forearm (ICPC: L72, ICD-9: 813, 814, ICD-10: S52), clinical spine (ICPC: L76.06, ICD-9: 805, 806, ICD-10: S12.0-S12.2, S12.7, S22.0, S22.1, S32.0-S32.2), humerus (ICPC: L74.04, ICD-9: 812, ICD-10: S42.2-S42.4, S42.7), and the composite category of MOF as defined by the WHO FRAX® algorithm (first of hip, forearm, clinical spine, or humerus). All patients were followed from the index date which was set at one year after start of valid data collection until either the date of right censoring (end date of valid data collection, end of the study period by 31 December 2011, or date of death) or the date of first fracture, whichever came first. The start and end date of valid data collection were respectively the first and last date where data was available in all data sources. This was done separately for each fracture category (hip, forearm, clinical spine, humerus, and the composite category MOF). Patients who sustained a prior fracture within the same category before the index date were excluded from the analyses. When a patient had sustained several fractures within the same category during follow-up, only the first fracture was counted for the calculation of incidence rates.

### Statistical analyses

Age-and-sex-specific incidence rates (number of fractures / 10,000 person years) were calculated by dividing the total number of fractures in that specific age-and sex- group by the total number of person years in that group and their 95% Confidence Intervals (95% CIs) were calculated [18]. This was done for 5-year age-categories over the period of valid data collection from 2002 up to 2011 and was reported from the age of 50 years. Age-standardized fracture rates and their 95% CIs were estimated by the direct method using the age-and-sex-structure of the Dutch population ≥50 years in 2008 [19]. Analyses were done separately for each fracture category. Finally, we compared observed age-and-sex-specific MOF rates to those predicted by the standardized incidence ratio (SIR; 95% CI). Predicted MOF rates were calculated by multiplying observed hip fracture rates with equal age-and-sex-specific incidence rate ratios of first hip fracture to first MOF as were observed in Malmö, Sweden which were previously used to calibrate the Dutch FRAX® algorithm for MOF risk ([11], Johansson personal communication). Analyses were performed using SAS statistical software, version 9.2 (SAS Institute, Inc., Cary, NC, USA).

## Results

A total of 5373 women aged 50 years and over sustained at least one MOF over 795,133 person-years of follow-up, in contrast to 1959 men over 810,052 person-years of follow-up. Table 1 shows age-and sex-specific incidence rates of first MOF, as well as the incidence of fractures of the hip, forearm, clinical spine, and humerus separately. Fractures of the forearm were the most dominant fracture type in the youngest age categories and hip fractures in the oldest age categories. For women at the age of 50–54 years, 9.6% of MOFs were hip fractures, as compared to 67.5% among those aged 90 years and older. A similar distribution for hip fracture was observed for men. With increasing age, there was a rise in incidence for all fracture categories in both men and women. The lowest incidence of MOF was observed for those 50–54 years (women: 22.2/10,000 person-years, men: 15.0/10,000 person-years) and the highest for those older than 90 years (women: 361.4/10,000 person-years, men: 166.7/10,000 person-years).

**Table 1:** Age- and sex-specific incidence rates (per 10,000 person years) of major osteoporotic fracture.

	Hip			Forearm			Clinical spine			Humerus			MOF*		
	N	IR	95% CI	N	IR	95% CI	N	IR	95% CI	N	IR	95% CI	N	IR	95% CI
**Women**															
50–54	35	2.1	1.4–2.8	229	13.9	12.1–15.7	40	2.4	1.7–3.2	65	3.9	3.0–4.9	363	22.2	19.9–24.5
55–59	47	3.0	2.1–3.8	352	22.6	20.2–24.9	57	3.6	2.7–4.6	115	7.3	6.0–8.7	552	35.6	32.6–38.6
60–64	94	6.7	5.4–8.1	417	30.0	27.2–32.9	98	7.0	5.6–8.4	145	10.4	8.7–12.0	717	52.2	48.4–56.0
65–69	103	9.3	7.5–11.1	387	35.3	31.8–38.8	95	8.6	6.8–10.3	144	13.0	10.9–15.1	682	63.2	58.4–67.9
70–74	178	19.3	16.5–22.1	366	40.1	36.0–44.2	129	13.9	11.5–16.3	139	15.0	12.5–17.5	763	85.3	79.3–91.4
75–79	254	35.6	31.2–40.0	337	47.4	42.4–52.5	132	18.3	15.2–21.4	141	19.6	16.4–22.8	795	115.8	107.8–123.9
80–84	330	72.3	64.5–80.1	235	51.0	44.5–57.6	129	27.6	22.8–32.3	117	25.0	20.5–29.6	729	167.4	155.2–179.5
85–89	263	116.5	102.4–130.6	103	44.4	35.8–52.9	80	34.0	26.5–41.4	96	40.9	32.7–49.0	477	223.6	203.5–243.7
90+	199	229.1	197.3–261.0	63	68.1	51.3–84.9	45	48.1	34.1–62.2	27	28.9	18.0–39.7	295	361.4	320.2–402.6
**Men**															
50–54	36	2.1	1.4–2.7	137	7.8	6.5–9.1	49	2.8	2.0–3.6	42	2.4	1.7–3.1	261	15.0	13.2–16.8
55–59	66	3.8	2.9–4.8	141	8.2	7.0–9.6	67	3.9	3.0–4.8	47	2.7	1.9–3.5	307	18.0	16.0–20.0
60–64	63	4.0	3.0–5.0	136	8.7	7.3–10.2	71	4.6	3.5–5.6	49	3.1	2.3–4.0	305	19.7	17.5–22.0
65–69	51	4.3	3.1–5.5	79	6.7	5.2–8.2	60	5.1	3.8–6.4	35	3.0	2.0–3.9	216	18.5	16.0–20.9
70–74	95	10.6	8.5–12.7	59	6.6	4.9–8.3	68	7.6	5.8–9.4	31	3.5	2.2–4.7	246	27.7	24.3–31.2
75–79	112	18.5	15.1–22.0	47	7.7	5.5–10.0	68	11.2	8.6–13.9	23	3.8	2.2–5.3	240	40.1	35.0–45.2
80–84	121	38.9	32.0–45.8	27	8.6	5.3–11.8	53	16.8	12.3–21.4	13	4.1	1.9–6.4	204	66.4	57.3–75.5
85–89	82	71.7	56.2–87.2	15	12.8	6.3–19.3	26	22.2	13.7–30.8	14	11.9	5.7–18.2	129	114.7	94.9–134.4
90+	37	117.7	79.8–155.6	5	15.4	1.9–28.8	8	24.7	7.6–41.8	5	15.4	1.9–28.8	51	166.7	120.9–212.4

Abbreviations: IR; incidence rate, 95% CI; 95% Confidence Interval, MOF; major osteoporotic fracture

* Includes first fracture of the hip, clinical spine, humerus, or forearm according to the FRAX® definition.

Table 2 shows the age-standardized incidence rates for men and women for the composite of MOF as well as for the MOF categories separately. MOF incidence (per 10,000 person-years) in men and women ≥ 50 years of age was estimated at 25.9 (95% CI: 24.7–27.0) and 77.0 (95% CI: 74.9–79.1) respectively. These numbers were 9.3 (95% CI: 8.6–10.0) and 24.0 (95% CI: 22.8–25.2) for hip fracture.

**Table 2:** Incidence rates (per 10,000 person years) of major osteoporotic fractures standardized to the Dutch population.

	Men (≥ 50 years)			Women (≥ 50 years)		
Fracture type	No. of fractures	IR	95% CI	No. of fractures	IR	95% CI
MOF *	1959	25.9	24.7–27.0	5373	77.0	74.9–79.1
Hip	663	9.3	8.6–10.0	1503	24.0	22.8–25.2
Forearm	646	8.0	7.4–8.6	2489	31.9	30.7–33.2
Clinical spine	470	6.0	5.5–6.6	805	11.1	10.3–11.8
Humerus	259	3.3	2.9–3.7	989	13.1	12.3–13.9

Abbreviations: IR; incidence rate, 95% CI; 95% Confidence Interval

* Includes first fracture of the hip, clinical spine, humerus, or forearm according to the FRAX® definition.

Table 3 shows observed and predicted age-and-sex-specific incidence rates of MOF. Among women, the observed incidence of MOF was significantly higher than predicted over a wide age-range (55–84 years). This difference was highest at the age of 65–69 years (SIR 1.50; 95% CI: 1.39–1.62). In men, the predicted incidence rates agreed reasonably well with those observed, but a significantly higher MOF rate was observed for those 50–54 years (SIR 1.63) and 65–69 years (SIR 1.22). Among the oldest old, the observed MOF rate was lower than predicted which was significant for men 85–89 years (SIR 0.79) and women ≥ 90 years (SIR 0.88).

**Table 3:** Age- and sex-specific observed incidence rates of major osteoporotic fracture as compared to those predicted by the imputation method.

	Hip _observed_		MOF _observed_		MOF _predicted_ *		
	N	IR	N	IR	N	IR	SIR (95% CI)
**Women**							
50–54	35	2.1	363	22.2	383	23.4	0.95 (0.85–1.05)
55–59	47	3.0	552	35.6	481	31.0	1.15 (1.05–1.25)
60–64	94	6.7	717	52.2	507	36.9	1.41 (1.31–1.52)
65–69	103	9.3	682	63.2	454	42.1	1.50 (1.39–1.62)
70–74	178	19.3	763	85.3	625	69.9	1.22 (1.14–1.31)
75–79	254	35.6	795	115.8	586	85.4	1.36 (1.26–1.45)
80–84	330	72.3	729	167.4	598	137.3	1.22 (1.13–1.31)
85–89	263	116.5	477	223.6	460	215.6	1.04 (0.95–1.13)
90+	199	229.1	295	361.4	335	410.1	0.88 (0.78–0.99)
**Men**							
50–54	36	2.1	261	15.0	160	9.2	1.63 (1.44–1.84)
55–59	66	3.8	307	18.0	280	16.4	1.10 (0.98–1.23)
60–64	63	4.0	305	19.7	338	21.9	0.90 (0.80–1.01)
65–69	51	4.3	216	18.5	177	15.1	1.22 (1.06–1.39)
70–74	95	10.6	246	27.7	243	27.4	1.01 (0.89–1.15)
75–79	112	18.5	240	40.1	230	38.4	1.05 (0.92–1.19)
80–84	121	38.9	204	66.4	198	64.6	1.03 (0.89–1.18)
85–89	82	71.7	129	114.7	163	144.8	0.79 (0.66–0.94)
90+	37	117.7	51	166.7	62	203.6	0.82 (0.61–1.08)

Abbreviations: IR; incidence rate, 95% CI; 95% Confidence Interval, MOF; major osteoporotic fracture (first fracture of the hip, clinical spine, humerus, or forearm, according to the FRAX® definition), SIR; standardized incidence ratio

*Predicted MOF rates were calculated by multiplying observed hip fracture rates by the age-and-sex-specific Swedish incidence rate ratios for first hip fracture to a first MOF. The expected number of MOF fractures were calculated by multiplying the predicted MOF rate to the total number of person-years in the corresponding age-and-sex-specific group.

## Discussion

This study provided age-and sex-specific incidence rates of hip and, for the first time, MOF as used in FRAX® in a large community-dwelling population in the Netherlands. Forearm fractures were the most dominant fracture type in the youngest age categories and hip fractures in the oldest age categories. The incidence rates of both hip and MOF increased with age for both genders. Among women 55–84 years, the observed incidence of MOF was significantly higher than predicted by the imputation method. In men, the imputation method performed reasonable. Finally, in the oldest old the observed MOF rates were significantly lower than predicted (≥ 90 years in women, and 85–89 years in men).

The general patterns of fracture incidence were in line with previous literature where incidence increased with age, was higher for women, and where forearm fractures were most dominant at younger age and hip fractures at older age [11, 20–22]. However, we found lower age-and-sex-specific incidence rates of vertebral and forearm fractures as compared to those reported by others [23–26]. The Rotterdam Study, a Dutch prospective cohort study, reported approximately 10-fold higher incidence rates of morphometrically ascertained vertebral fractures [24]. Incidence rates of forearm fractures were approximately 2-fold higher [26]. Although only one third [27] to one fourth [28] of all morphometric vertebral fractures come to clinical attention, a 10-fold lower incidence rate indicates substantial under reporting of vertebral fractures in general practitioner records. This finding is supported by a Spanish validation study where under-recording of vertebral and forearm fractures was high in general practitioner records (56% and 50%, respectively) when compared to a prospective cohort study [29].

Hip fracture rates were also lower when compared to a nationwide study that used hospital discharge records from 2004 to 2005, which was used to calibrate FRAX® to the Dutch population [10]. This may be related to a secular decline in hip fracture incidence that was reported in the Netherlands between 1996 and 2008 with a percentage annual change of -0.64% in women and -0.34% in men [13]. This study used nationwide data that was corrected for missing values by Statistics Netherlands. From the year 2005, Dutch hospitals were no longer required to record hospitalisations by ICD-codes and send them to the national registry. This has led to an increase in missing or non-linking records from 3.5% in 2002 to 14% in 2007 [10, 30]. Imputation resulted in missing’s ranging between 2.6% and 3.5% due to non-linking records over the same period. To overcome this limitation, we linked hospitalisations to general practitioner records but under recording may still have been present. A further explanation may be a difference in general health between the study population and the total population of the Netherlands. The present study only included community-dwelling individuals while the incidence of hip fracture has been reported to be 2 to 20-fold higher in institutionalized patients, depending on age and sex [31, 32]. Indeed, the Global Longitudinal Study of Osteoporosis in Women (GLOW, 2006–2013) that prospectively estimated hip fracture incidence in an international population-based community-dwelling population, found similar hip fracture rates (80–84 years: 70.0/10,000 person-years) [22, 33]. The higher observed incidence of MOF as compared to that predicted by the imputation method is in line with scarcely available evidence from other countries [22, 34, 35]. A Canadian study used hospitalisation and claims data to obtain the hip fracture/MOF incidence rate ratios over the period 2000–2007 [34]. The Canadian ratios were significantly higher than the Swedish ratios for women 55–74 years while this was only observed among men 55–59 years. An Icelandic study showed a significantly higher MOF incidence as compared to that predicted among women 60–69 years (33%) and among men 50–59 years (28%) [35]. Furthermore, although the total number of fractures was limited, the GLOW study similarly reported higher hip/MOF ratios than those reported in Sweden [22]. Any miscalibration of FRAX® will influence predicted absolute fracture risk, and subsequently the individual risk communication between the physician and the patient and the decision to prescribe anti-osteoporotic drugs. It may have a substantial impact on treatment decisions worldwide, since the online FRAX® tool is frequently used with over 13 million hits between 2011 and 2015 where the majority of the country-specific FRAX® tools incorporate imputed MOF rates due to lack of data. Specific treatment thresholds for FRAX® are not incorporated into Dutch guidelines, but several international guidelines (e.g. the USA) specifically state to initiate treatment above a certain threshold of FRAX® predicted absolute fracture risk. Indeed, a simulation study showed that a 20% underestimation in MOF risk resulted in a 50% decrease in the numbers categorized as needing treatment when the treatment threshold was set at 20% [36]. Apart from differences in geographic region, a possible explanation for the underestimation of MOF incidence by the imputation method is a secular change in fracture incidence, where the drop in MOF incidence proceeded more slowly than for hip fracture alone. The decline in hip fracture incidence in the Netherlands was greatest among the younger age categories (65–69: -23%, 70–79: -13.9%, 80–84: -5.4%) and among women [13]. Over the same time period, forearm fracture incidence declined, but less marked than at the hip, among younger women (60–69; -18.4%, 70–79; -5%) while rates remained stable among the elderly [37]. Vertebral fracture incidence has even increased in both Dutch men and women aged ≥ 65 years [23]. The slower decline in incidence of MOF as compared to the hip was similarly observed in the limited number of studies that evaluated secular trends for hip and non-hip major osteoporotic fractures, including Canada [21] and Iceland [20]. The reasons for the secular changes in fracture epidemiology remain poorly understood. It may be related to increased health and functional ability of the population [38, 39]. A change in frequency of risk factors for fracture such as physical activity, vitamin D insufficiency, and smoking status may all have contributed to changes in fracture risk. The increase in body mass index, which was reported worldwide [40], may also have reduced hip fracture risk. The implementation of anti-osteoporosis drug treatment or fall prevention programs could further have contributed to reduced fracture risk, but is unlikely to be fully responsible since the secular decline in hip fracture incidence initiated already before these measures. Furthermore, one should consider data quality when interpreting secular changes in fracture incidence. This includes knowledge of changes in the coding system, and of increases [41] or decreases in the rate of reporting. Finally, incidence rates may be influenced by the underlying study population which in turn is influenced by the way databases are being linked. For example, linkage of a community pharmacy-based cohort to hospitalisations, as used in this study, excluded the institutionalized population. The Dutch FRAX® algorithm was calibrated with higher hip fracture rates than observed in the present study [10]. The imputed MOF rates are therefore still equivalent or higher than the MOF rates from the present study, despite evidence for violation of the Swedish hip to MOF imputation method. We could not reliably calculate true age-and-sex-specific hip/MOF ratios as non-hip MOFs were likely under-recorded in our database. It is important to use other data sources to update the fracture epidemiology in the Netherlands and to confirm our results. An alternative for estimating fracture incidence is claims data. The Dutch VEKTIS database has nationwide coverage with complete fracture data. However, patient-specific data should be available since aggregated age-and-sex-specific data leads to inability to adjust incidence for previous or subsequent fractures. This would result in substantially higher IRs and thus overestimation of MOF risk when these rates were used to calibrate FRAX®, as was observed in an Icelandic study [35]. A further drawback includes the lag time of up to two years in registration of claims data. A second alternative may be linkage of general practitioner records to emergency department records where all non-hip fractures enter the system. Linkage to GP records then would enable calculation of incidence rates on a patient-level. Our study had additional limitations. Due to the probabilistic linkage process we may have missed fractures. In addition, the source population was not fully representative of the total population and results can therefore not be extrapolated to the institutionalized population. Third, fractures were ascertained from administrative data which is less reliable than radiographic or medical chart review. However, a high positive predictive value (>90%) has been shown for hip, vertebral, and forearm fractures in general practitioner records [29, 42] and for hip fracture in the PHARMO Database Network [17]. Finally, a more general limitation of FRAX® includes that many other fracture sites than those included in FRAX® have been associated with osteoporosis [22, 43]. Their neglect may underestimate true fracture risk. A major strength of this study included the linkage of longitudinal general practitioner, hospitalization and mortality records for a reasonably large part of the Netherlands. It allowed anonymized person-specific follow-up to estimate the incidence of MOF as used in FRAX®. The Rotterdam Study [24, 26] also estimated fracture incidence at a patient-level, but not for MOF as used in FRAX® and extrapolation of results may have been hampered as this study was performed in the region of Rotterdam only. In conclusion, observed MOF incidence was higher than predicted by the imputation method for women over a wide age range while there was reasonable agreement among men. Despite evidence for invalidity of the imputation method to estimate MOF incidence, the Dutch FRAX® algorithm currently incorporates equivalent or higher incidence rates for MOF due to higher hip fracture rates. As miscalibration may affect treatment decisions, there is a need for confirmation of results in another data-source. Until then, the Dutch FRAX® output should be interpreted with caution.
